# No Relevant Associations Between Markers of Smoking Behaviour and Plasma Progesterone Concentrations: Findings From a Sex‐Stratified Cohort Study

**DOI:** 10.1111/adb.70071

**Published:** 2025-08-08

**Authors:** Julia Gihl, Norman Zacharias, Sabine Hoffmann, Norbert Thürauf, Gerd Schaller, Georg Winterer, Anne Koopmann, Falk Kiefer, Johannes Kornhuber, Christiane Mühle, Bernd Lenz

**Affiliations:** ^1^ Department of Psychiatry and Psychotherapy Friedrich‐Alexander‐Universität Erlangen‐Nürnberg (FAU), and Universitätsklinikum Erlangen Erlangen Germany; ^2^ Department of Otolaryngology, Head and Neck Surgery Charité – Universitätsmedizin Berlin Berlin Germany; ^3^ Department of Addictive Behavior and Addiction Medicine, Central Institute of Mental Health (CIMH), Medical Faculty Mannheim Heidelberg University Mannheim Germany; ^4^ Department of Psychiatry Hospital of Bressanone (SABES‐ASDAA) Bressanone Italy; ^5^ Campus Virchow and Campus Mitte, Charité Universitätsmedizin Berlin Berlin Germany

**Keywords:** addiction, cigarette smoking, progesterone, sex hormones, sex steroids, substance use disorder, tobacco use disorder

## Abstract

Cigarette smoking is a prevalent and critical global health issue, with inconsistent findings for its effects on endogenous progesterone concentrations. This large multicentre study investigated the associations between various markers of smoking behaviour and plasma progesterone concentrations using a sex‐segregating approach. We studied 747 males aged 18–65 years and 158 peri−/postmenopausal females aged 50–65 years and assessed differences in plasma progesterone concentrations between smokers and never‐smokers and associations of plasma progesterone concentrations with the Fagerström Test for Nicotine Dependence (FTND) score, cigarette pack years, age at onset of regular smoking, number of cigarettes smoked daily, exhaled carbon monoxide (CO), plasma cotinine and the Questionnaire of Smoking Urges (QSU) score. In models adjusted for age, body mass index (BMI), years of education, Alcohol Use Disorder Identification Test (AUDIT) scores, intake of any medication and study centre, and after correction for multiple hypothesis testing, there were no significant differences in plasma progesterone concentrations between smokers and never‐smokers, and no significant associations between any of the mentioned markers of smoking behaviour and plasma progesterone concentrations in either males or females. The results suggest that smoking behaviour has no substantial effect on plasma progesterone concentrations and is not an important confounder in studies investigating progesterone.

## Graphical abstract

This multicentre study examined the relationship between smoking behaviour and plasma progesterone concentrations in 747 males and 158 peri/postmenopausal females. In sex–separated, covariate–adjusted analyses, no relevant differences in progesterone were found between smokers and never–smokers, nor were there associations of progesterone with biological or behavioural markers of smoking. These findings suggest that smoking does not substantially affect plasma progesterone concentrations.

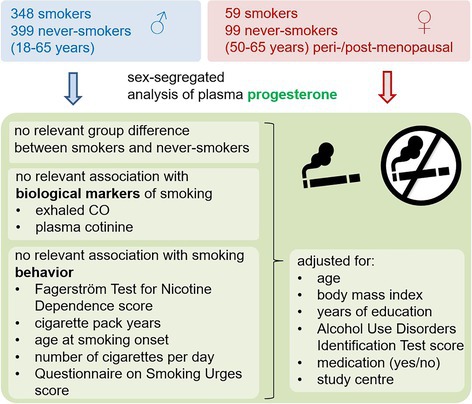

## 1 Introduction

Cigarette smoking stands as one of the most significant preventable causes of mortality worldwide. It results in over eight million annual deaths and several million lost disability adjusted life years per year according to the World Health Organization's (WHO) latest report on the global tobacco epidemic [1]. The substances contained in cigarette smoke, including nicotine, polycyclic aromatic hydrocarbons, nitrosamines and carbon monoxide (CO), have been proven to exert pro‐inflammatory and carcinogenic effects [2]. Furthermore, it is hypothesized that cigarette smoking is linked to variations in sex steroid concentrations. Previous research has revealed that smoking is associated with higher androgen concentrations in both males [3, 4, 5] and postmenopausal females [6, 7, 8] and with higher oestrogen concentrations in males [9, 10].

However, for the sex steroid progesterone, there is a significant research gap regarding the effects of smoking on its plasma concentrations. Progesterone and its neurosteroid derivatives modulate both the dopamine system, which influences reward and addictive behaviours, and the GABA system, which exerts anxiolytic and sedative effects [11]. Consistent with its neurobiological role in addiction, an association between progesterone and other substance use disorders (SUDs) has been established. Notably, a well‐documented link exists between progesterone levels and alcohol consumption in females [12, 13, 14, 15]. Even in males, emerging evidence suggests that progesterone may play a dynamic role in alcohol use behaviours, as a recent study found short‐term fluctuations in progesterone concentrations in males with alcohol use disorder (AUD) [16]. These links between progesterone, the neurobiological reward system and SUDs underscore its significance and highlight the need for further investigation.

Regarding smoking and progesterone, it is noteworthy that to our knowledge, no previous studies have included males, despite their higher smoking prevalence (36.7% compared with 7.8% in females, according to the WHO [17]). This leaves a significant gap in understanding the effects of smoking behaviours on plasma progesterone concentrations in the male sex.

The only few identified studies—primarily on premenopausal females—have yielded inconsistent results. Some studies have reported lower progesterone concentrations in smokers compared with non‐smokers during the follicular phase [18] or unknown cycle phase [19], while others have found higher concentrations [20, 21, 22], and some have demonstrated no differences [23, 24]. Very few studies investigated postmenopausal females and found either higher progesterone concentrations in smokers [6, 7] or reported no significant differences [25, 26].

The current literature is also limited by small sample sizes (e.g., *N* = 16 [20]) and imbalanced groups (e.g., 16 smokers vs. 243 non‐smokers [24]), which raises concerns about the robustness of the findings. The evaluation of smoking behaviour in these studies was based on self‐reported data, with varying criteria for categorizing smokers and non‐smokers [18, 19, 20, 21, 22, 23, 24, 26], which limits comparability. Furthermore, there was a lack of consideration of objective biological markers of smoking.

It is therefore imperative that more rigorously designed studies are conducted to elucidate the relationship between smoking and plasma progesterone concentrations, particularly because smoking should be considered a confounding factor in studies of SUDs. Tobacco consumption exhibits a high global prevalence of 20% [1], and tobacco use disorder (TUD) is markedly comorbid with other SUDs [27, 28, 29, 30, 31]. Individuals with SUDs are two to four times more likely to smoke than the general population [31].

The primary objective of this study was to investigate the association between smoking behaviour and plasma progesterone concentration using a sex‐segregating approach [32]. The groups consisted of males and peri‐/postmenopausal females to avoid bias by fluctuating hormone concentrations across the menstrual cycle. The specific aims were as follows:

1. To identify group differences in plasma progesterone concentrations between smokers and never‐smokers in sex‐segregated groups.
2. To identify cross‐sectional associations of plasma progesterone concentrations with various short‐ and long‐term markers of exposure to smoking, i.e., cigarette pack years, exhaled CO, plasma cotinine and markers of smoking behaviour, i.e., age of onset of regular smoking, number of cigarettes smoked daily, the Fagerström Test for Nicotine Dependence (FTND) score and the Questionnaire of Smoking Urges (QSU) score.

## 2 Materials and Methods

### 2.1 Study Description

This project was based on data and plasma samples from the Priority Program (Schwerpunktprogramm) SPP1226: ‘Nicotine – Molecular and Physiological Effects in CNS’ [33]. A primary sample of 55 000 subjects was randomly selected from the residents' register at seven psychiatric university hospital locations between 2007 and 2009 and contacted by letter. Participants received a monetary reward of €50 for their participation. All participants underwent a multistep screening and recruitment procedure consisting of clinical examinations, standardized clinical interviews, drug screenings, alcohol testing and exhaled CO measurement. Inclusion criteria were age 18–65 years, smokers with a minimum of one cigarette a day or never‐smokers with a maximum of 20 cigarettes in their lifetime, and German descent. Exclusion criteria were former smokers without current consumption, substance abuse within the last 6 months except for tobacco, any medical condition that could affect sex steroid concentrations, in particular pregnancy, current substance dependency except for tobacco (according to DSM‐IV), psychiatric comorbidities within the last 6 months (according to Axis‐1‐DSM‐IV), central nervous system diseases within the lifespan and any medication that could affect the central nervous system [33]. Consequently, 2396 participants were included. Blood plasma samples were collected during recruitment, processed following a standardized operating procedure and stored at −20°C until analysis of progesterone and cotinine.

### 2.2 Psychometric Scales and Assessment of Smoking Behaviour

Diagnosis and exclusion of psychiatric comorbidities was based on the Structured Clinical Interview (SCID‐I) from the Diagnostic and Statistical Manual of Mental Disorders (DSM‐IV). The German version of the FTND was employed to assess cigarette dependence [34] and the German version of the QSU [35] to quantify nicotine cravings. To record current cigarette consumption, the number of cigarettes currently smoked per day was surveyed. To assess cumulative cigarette exposure, cigarette pack years were calculated, with one pack‐year defined as smoking 20 cigarettes per day for one year, based on participants' reported daily cigarette consumption and duration of smoking.

### 2.3 Quantitative Analysis of Progesterone and Biological Markers

Progesterone was quantified in EDTA plasma samples by competitive enzyme‐linked immunosorbent assays (Progesterone ELISA AMP 30‐E2700, Asbach Medical Products GmbH, Obrigheim, Germany) with a 25 μL sample volume in duplicates and a standard range from 0.15 to 40 ng/mL according to the manufacturer's instructions. All samples were analysed in nine batches by the same, blinded staff. Achieved intra‐ and inter‐assay coefficients of variation were 2.8% and 9.5%, respectively. Blood samples and progesterone data were available from 1977 individuals.

To quantify nicotine consumption using a biological marker, plasma cotinine and exhaled CO were determined. Plasma concentrations of cotinine were measured by radioimmunoassay. The intra‐assay and inter‐assay coefficients of variation were both below 7% [33]. Exhaled CO concentrations were measured in the morning before cigarette consumption [36].

### 2.4 Statistical Analysis

After exclusion of individuals who reported taking medication with effects on the sex steroid system (*N* = 195), females younger than 50 years (= median age at cessation of menses in females in Germany [37], *N* = 769), subjects with missing data for body mass index (BMI, *N* = 6), years of education (*N* = 10) or AUDIT score (*N* = 12), and plasma progesterone concentrations exceeding the median ± 1.5 interquartile ranges (*N* = 1 male below, *N* = 59 males above, *N* = 20 females above limit), we included 905 subjects in our investigation.

In a first step, we used analysis of covariance separated for males and peri‐/postmenopausal females to study the associations of plasma progesterone concentrations with smoking status (smoker versus never‐smoker), FTND score, cigarette pack years, age of onset of regular smoking, number of cigarettes smoked daily, exhaled CO, plasma cotinine and QSU score in separate models. These were adjusted for age, BMI, years of education, AUDIT score, intake of any medication (yes = 2 versus no = 1) and study centre. In a second step, we computed these analyses again in the following similarly large age‐specific subgroups of males: 18–27 years (*N* = 259), 28–43 years (*N* = 239), 44–65 years (*N* = 249).

We analysed the data using SPSS (IBM SPSS for Windows 29.0; SPSS Inc., Chicago, IL, USA) and employed Student's *t*‐test and χ^2^ test for the sample description. Alpha was set at 0.05 (two‐sided). Tables 2, 3, 4, 5, 6, 7, 8, 9 show B values only for nominally significant parameters. To interpret the eight primary hypotheses in each sex, we used a Bonferroni corrected threshold of 0.05/16 = 0.003.

## 3 Results

### 3.1 Cohort Characteristics

In total, we analysed data from 905 subjects, containing 747 males and 158 peri‐/postmenopausal females (Table 1). Age was higher in smokers versus never‐smokers in males and lower in smokers versus never‐smokers in females.

**TABLE 1:** Sample characteristics.

	*N*	Mean/RF	SD	*N*	Mean/RF	SD	T/χ^2^	*p*
	Smokers			Never‐smokers				
Males 18–65 years								
Age (years)	348	39.14	13.05	399	35.16	12.85	4.196	< 0.001
BMI (kg/m^2^)	348	25.80	4.47	399	25.08	3.73	2.356	0.019
Years of education	348	14.89	2.96	399	16.10	2.79	−5.750	< 0.001
AUDIT score	348	5.66	3.84	399	3.93	2.87	6.852	< 0.001
% intake of medication	348	31.03		399	34.09		0.787	0.375
FTND score	341	3.19	2.65					
Cigarette pack years	346	20.05	21.28					
N cigarettes/day	348	15.44	10.91					
CO exhaled (ppm)	323	14.55	10.50	381	1.86	1.67	21.488	< 0.001
Cotinine (ng/mL)	347	118.86	103.32	398	5.11	47.86	18.824	< 0.001
QSU score	339	102.43	37.98					
Age at smoking onset (years)	346	16.29	3.45					
Plasma progesterone (ng/mL)	348	0.421	0.139	399	0.420	0.133	0.103	0.918
Females 50–65 years								
Age (years)	59	55.56	4.37	99	57.67	4.33	−2.950	0.004
BMI (kg/m^2^)	59	25.34	4.44	99	25.87	4.96	−0.684	0.495
Years of education	59	13.69	2.73	99	14.27	2.90	−1.227	0.221
AUDIT score	59	3.29	3.34	99	2.66	2.41	1.374	0.171
% intake of medication	59	55.93		99	70.71		3.556	0.059
FTND score	56	3.09	2.38					
Cigarette pack years	59	27.18	16.39					
*N* cigarettes/day	59	14.47	8.23					
CO exhaled (ppm)	55	13.98	12.46	97	1.26	1.49	7.541	< 0.001
Cotinine (ng/mL)	59	122.03	120.53	99	9.64	66.72	6.587	< 0.001
QSU score	55	101.58	44.17					
Age at smoking onset (years)	59	18.25	5.63					
Plasma progesterone (ng/mL)	59	0.235	0.085	99	0.226	0.084	0.655	0.513

Abbreviations: AUDIT, Alcohol Use Disorder Identification Test; BMI, body mass index; FTND, Fagerström Test for Nicotine Dependence; *N* cigarettes/day, N cigarettes smoked per day; ppm, parts per million; QSU, Questionnaire of Smoking Urges.

BMI was higher in smokers versus never‐smokers in males. Years of education were lower in smokers versus never‐smokers in males. The AUDIT score was higher in smokers versus never‐smokers in males.

According to their smoking status, exhaled CO and cotinine were higher in smokers versus never‐smokers in both males and females.

### 3.2 Difference in Plasma Progesterone Concentrations Between Smokers and Never‐Smokers

Considering the adjusted significance threshold of 0.003 due to multiple hypothesis testing, the group allocation (i.e., smokers versus never‐smokers) was not significantly associated with plasma progesterone concentrations in either males or females (Table 2).

**TABLE 2:** Univariate analysis of covariance to predict plasma progesterone concentrations by *smoking status*.

	Males 18–65 years (*N* = 747)				Females 50–65 years (*N* = 158)			
	B	F	*p*	Partial η^2^	B	F	*p*	Partial η^2^
Smoking status	0.022	4.680	0.031	0.006		0.035	0.852	0.000
Age	−0.004	110.177	< 0.001	0.131	−0.004	5.824	0.017	0.039
BMI	−0.007	38.302	< 0.001	0.050		0.512	0.475	0.004
Years of education		0.848	0.358	0.001		2.677	0.104	0.018
AUDIT score		2.646	0.104	0.004		0.122	0.727	0.001
Medication		0.166	0.684	0.000		1.085	0.299	0.007
Study centre		1.705	0.117	0.014		1.519	0.176	0.059

*Note:* Smoking status: 1 = smoker vs. 2 = never‐smoker.

Abbreviations: AUDIT, Alcohol Use Disorder Identification Test; BMI, body mass index.

In contrast, age was linked to plasma progesterone concentrations in males and in peri‐/postmenopausal females while BMI was related to plasma progesterone concentrations in males.

### 3.3 Associations of Smoking Behaviours With Plasma Progesterone Concentrations

None of the investigated smoking behaviours was significantly associated with plasma progesterone concentrations in males or peri‐/postmenopausal females (Tables 3, 4, 5, 6, 7, 8, 9). As for smoking status, age and BMI were consistently associated with plasma progesterone concentrations in males across all analyses (Table 3, 4, 5, 6, 7, 8, 9).

**TABLE 3:** Univariate analysis of covariance to predict plasma progesterone concentrations by *FTND score*.

	Males 18–65 years (*N* = 341)				Females 50–65 years (*N* = 56)		
	B	F	*p*	Partial η^2^	F	*p*	Partial η^2^
FTND		0.378	0.539	0.001	0.085	0.772	0.002
Age	−0.004	58.389	< 0.001	0.151	0.081	0.778	0.002
BMI	−0.008	23.794	< 0.001	0.068	0.002	0.967	0.000
Years of education		1.975	0.161	0.006	1.425	0.239	0.031
AUDIT score		1.275	0.260	0.004	0.039	0.845	0.001
Medication		0.699	0.404	0.002	0.006	0.939	0.000
Study centre		1.882	0.083	0.033	0.933	0.469	0.096

Abbreviations: AUDIT, Alcohol Use Disorder Identification Test; BMI, body mass index; FTND, Fagerström Test for Nicotine Dependence.

**TABLE 4:** Univariate analysis of covariance to predict plasma progesterone concentrations by *cigarette pack years*.

	Males 18–65 years (*N* = 346)				Females 50–65 years (*N* = 59)		
	B	F	*p*	Partial η^2^	F	*p*	Partial η^2^
Pack years		0.136	0.712	0.000	0.004	0.948	0.000
Age	−0.004	42.326	< 0.001	0.113	0.146	0.704	0.003
BMI	−0.008	24.512	< 0.001	0.069	0.007	0.934	0.000
Years of education		2.269	0.133	0.007	1.605	0.212	0.033
AUDIT score		0.995	0.319	0.003	0.002	0.961	0.000
Medication		0.571	0.450	0.002	0.002	0.968	0.000
Study centre		1.966	0.070	0.034	1.052	0.399	0.101

Abbreviations: AUDIT, Alcohol Use Disorder Identification Test; BMI, body mass index; Pack years = cigarette pack years calculated as (number of packs [20 cigarettes] smoked per day) × (number of years smoked).

**TABLE 5:** Univariate analysis of covariance to predict plasma concentrations by *age at smoking onset*.

	Males 18–65 years (*N* = 346)				Females 50–65 years (*N* = 59)		
	B	F	*p*	Partial η^2^	F	*p*	Partial η^2^
Smoking onset		0.135	0.714	0.000	0.369	0.547	0.008
Age	−0.004	60.296	< 0.001	0.153	0.263	0.610	0.006
BMI	−0.008	24.526	< 0.001	0.069	0.002	0.963	0.000
Years of education		2.242	0.135	0.007	1.210	0.277	0.025
AUDIT score		0.980	0.323	0.003	0.005	0.946	0.000
Medication		0.527	0.468	0.002	0.001	0.975	0.000
Study centre		1.976	0.068	0.034	1.012	0.421	0.097

Abbreviations: AUDIT, Alcohol Use Disorder Identification Test; BMI, body mass index; smoking onset, age at smoking onset (years).

**TABLE 6:** Univariate analysis of covariance to predict plasma concentrations by *number of cigarettes per day*.

	Males 18–65 years (*N* = 348)				Females 50–65 years (*N* = 59)		
	B	F	*p*	Partial η^2^	F	*p*	Partial η^2^
*N* cigarettes/day		0.577	0.448	0.002	0.106	0.746	0.002
Age	−0.004	61.027	< 0.001	0.154	0.064	0.801	0.001
BMI	−0.008	24.580	< 0.001	0.068	0.008	0.927	0.000
Years of education		2.179	0.141	0.006	1.922	0.172	0.039
AUDIT score		0.982	0.322	0.003	0.000	0.984	0.000
Medication		0.603	0.438	0.002	0.004	0.948	0.000
Study centre		1.889	0.082	0.033	0.954	0.455	0.092

Abbreviations: AUDIT, Alcohol Use Disorder Identification Test; BMI, body mass index; *N* cigarettes/day, *N* cigarettes smoked per day.

**TABLE 7:** Univariate analysis of covariance to predict plasma progesterone concentrations by *exhaled CO*.

	Males 18–65 years (*N* = 323)				Females 50–65 years (*N* = 55)		
	B	F	*p*	Partial η^2^	F	*p*	Partial η^2^
Exhaled CO		1.498	0.222	0.005	0.503	0.482	0.012
Age	−0.004	61.863	< 0.001	0.166	0.031	0.860	0.001
BMI	−0.008	23.306	< 0.001	0.070	0.000	0.996	0.000
Years of education		2.147	0.144	0.007	1.744	0.194	0.039
AUDIT score		1.248	0.265	0.004	0.038	0.847	0.001
Medication		0.404	0.525	0.001	0.053	0.818	0.001
Study centre		1.809	0.097	0.034	1.516	0.205	0.150

Abbreviations: AUDIT, Alcohol Use Disorder Identification Test; BMI, body mass index.

**TABLE 8:** Univariate analysis of covariance to predict plasma progesterone concentrations by *cotinine*.

	Males 18–65 years (*N* = 347)				Females 50–65 years (*N* = 59)		
	B	F	*p*	Partial η^2^	F	*p*	Partial η^2^
Cotinine		3.568	0.060	0.011	0.024	0.878	0.001
Age	−0.004	65.062	< 0.001	0.163	0.185	0.669	0.004
BMI	−0.007	23.928	< 0.001	0.067	0.003	0.955	0.000
Years of education		2.291	0.131	0.007	1.397	0.243	0.029
AUDIT score		1.307	0.254	0.004	0.008	0.931	0.000
Medication		0.530	0.467	0.002	0.001	0.970	0.000
Study centre		1.888	0.082	0.033	1.090	0.378	0.104

Abbreviations: AUDIT, Alcohol Use Disorder Identification Test; BMI, body mass index.

**TABLE 9:** Univariate analysis of covariance to predict plasma progesterone concentrations by *QSU score*.

	Males 18–65 years (*N* = 339)				Females 50–65 years (*N* = 55)		
	B	F	*p*	Partial η^2^	F	*p*	Partial η^2^
QSU score		0.027	0.870	0.000	0.371	0.546	0.009
Age	−0.004	56.934	< 0.001	0.149	0.007	0.935	0.000
BMI	−0.008	23.382	< 0.001	0.067	0.000	0.995	0.000
Years of education		2.083	0.150	0.006	2.285	0.138	0.050
AUDIT score		1.140	0.286	0.003	0.000	0.983	0.000
Medication		0.648	0.421	0.002	0.036	0.850	0.001
Study centre		1.827	0.093	0.033	0.774	0.574	0.083

Abbreviations: AUDIT, Alcohol Use Disorder Identification Test; BMI, body mass index; QSU, Questionnaire of Smoking Urges.

### 3.4 Age Group‐Specific Analyses

To account for evidence suggesting an age‐related decline in plasma progesterone concentrations in males [38], a post hoc analysis was conducted across age‐stratified groups (Tables S1–S9). Consistent with previous models, no significant associations were found between plasma progesterone concentrations and group status (smokers vs. never‐smokers) or any specific smoking behaviour.

## 4 Discussion

The objective of this study was to identify cross‐sectional associations between various proxies of cigarette smoking behaviour and plasma progesterone concentrations. After adjusting the significance threshold for multiple hypothesis testing, no significant differences in progesterone concentrations were observed between smokers and never‐smokers in either males or peri−/postmenopausal females. While a nominally significant difference was found in the model for the total male group (*p* = 0.031), the effect size was small (partial η^2^ = 0.006) and should be interpreted with caution. Furthermore, no significant relationships were identified between the investigated psychometric and biological markers of smoking behaviour and plasma progesterone concentrations in males or peri−/postmenopausal females.

Overall, the strongest effect size among the non‐significant associations between smoking behaviours and plasma progesterone concentrations was observed for exhaled CO in peri‐/postmenopausal females, with a partial η^2^ of 0.012, corresponding to a small effect size. Taken together, the results of this study suggest that smoking does not exert a relevant influence on plasma progesterone concentrations in males or peri‐/postmenopausal females. A previous study [25] further supports this conclusion, reporting similar results in a subgroup of postmenopausal females. In that study, progesterone concentrations were measured in smokers and non‐smokers of comparably sized groups (36 smokers, 40 non‐smokers), with participants matched for age and BMI. The authors conducted an analysis of covariance, adjusting for age and BMI as continuous covariates—an approach that enhances comparability with the present study. These adjustments strengthen the validity of the findings, given the strong associations between age, BMI and smoking behaviour [39, 40, 41, 42].

Previous studies have reported conflicting results regarding the association between smoking and progesterone concentrations [6, 7, 25, 26], reflecting substantial variability in effect estimates. A meta‐analysis focusing on premenopausal females [43] reported an *I* ^2^ statistic ranging from 60% to 100%, indicating high heterogeneity among these studies. This variability underscores the limited reliability of existing evidence on this topic. The observed heterogeneity is likely attributable to the lack of adjustment for key confounding variables influencing smoking behaviour—adjustments that were incorporated in the current study.

Although previous studies commonly adjusted for age, few accounted for years of education [7, 19, 20, 21, 22, 23, 24, 25, 26] or medications that might influence sex steroid concentrations [7, 18, 19, 21, 23, 24, 25, 26]. Moreover, several studies did not include BMI as a covariate [18, 20, 23]. In the present analysis, both higher age and BMI were significantly associated with lower plasma progesterone concentrations in males. The age‐related decline in progesterone may reflect reduced steroidogenesis with aging [38]. Similarly, elevated BMI may disrupt hormonal balance through obesity‐induced alterations in hypothalamic–pituitary–gonadal axis function, contributing to decreased plasma sex steroid concentrations [44]. These findings underscore the importance of adjusting for age and BMI as relevant covariates when analysing plasma progesterone concentrations.

Notably, none of the previous studies adjusted for AUDIT scores [7, 18, 19, 20, 21, 22, 23, 24, 25, 26]. The results of research conducted on both male and female rodents, as well as humans, demonstrate that acute and chronic alcohol exposure is associated with a moderate to substantial reduction in progesterone concentrations in both sexes (and a notable elevation in estradiol concentrations) [12, 13, 14, 15, 45, 46, 47]. Conversely, elevated ratios of progesterone to estradiol have been suggested to confer a protective effect against problematic alcohol consumption [16] and higher progesterone concentrations correlate with lower craving in postmenopausal females with AUD [48]. Therefore, it would be beneficial for future research to consistently adjust for alcohol use to better isolate the specific effects of smoking behaviour on plasma progesterone concentrations.

### 4.1 Strengths and Limitations

To our knowledge, this is the first study to investigate the effects of smoking behaviour on plasma progesterone concentrations in males. We analysed a large sample of smokers and never‐smokers with 747 male and 158 peri‐/postmenopausal female subjects across a wide age range at multiple locations in Germany. We here provide results from a study with high test power, which consistently reports null findings in contrast to contradictory findings from smaller studies.

We used models adjusted for the possible confounding variables of age, BMI, years of education, medication and AUDIT score, therefore achieving high discriminatory power for effects solely attributable to smoking. Notably, individuals with TUD in the past year have an adjusted odds ratio of 1.9–2.9 for also having AUD compared with the general population [30]. Similarly, smokers had higher AUDIT scores than never‐smokers in males in our study, highlighting the importance of adjustment for this variable.

Moreover, we examined numerous endpoints related to smoking behaviour, allowing a broader evaluation. Smoking behaviour was recorded both through self‐reports using the FTND and QSU questionnaires and via the objective biological markers. While most of the previous studies (except for [18, 21]) relied solely on self‐reports, which have been shown to be subject to recall bias and to have limited reliability [18], we minimized this bias by incorporating two biological measures: We assessed current smoking behaviour using short‐half‐life exhaled CO (t_1/2_ = 4.5–6 h [49]) and smoking over the preceding days via plasma cotinine (t_1/2_ = 10–30 h [49]). Unlike other studies, we compared smokers with strict never‐smokers (defined as having smoked fewer than 20 cigarettes in their lifetime), enhancing the discriminatory power between groups.

The plasma progesterone measurements used here are more stable against inter‐ and intraindividual as well as circadian fluctuations than saliva measurements [50]. A potential bias due to circadian fluctuations of plasma progesterone concentrations was minimized by taking all blood samples during predefined morning time windows [33].

### 4.2 Implications for Further Research

The results suggest that smoking is not a relevant confounder in studies investigating the relationship between SUDs and plasma progesterone concentrations. The importance is underlined by the high comorbidity of cigarette smoking and other SUDs [27, 28, 29]. In studies on SUDs, the groups of peri‐/postmenopausal females and males are highly prevalent [17], highlighting the importance of recruiting these groups for future research.

## Author Contributions

**Julia Gihl:** conceptualization, methodology, investigation, data curation, formal analysis, writing – original draft preparation, writing – review and editing. **Norman Zacharias:** resources, data curation. **Sabine Hoffmann:** formal analysis. **Norbert Thürauf:** resources. **Gerd Schaller:** writing – review and editing. **Georg Winterer:** resources. **Anne Koopmann:** resources. **Falk Kiefer:** resources, funding. **Johannes Kornhuber:** resources, funding. **Christiane Mühle:** conceptualization, methodology, investigation, data curation, formal analysis, project administration, supervision, writing – original draft preparation, writing – review and editing. **Bernd Lenz:** conceptualization, data curation, formal analysis, project administration, supervision, writing – original draft preparation, writing – review and editing. All authors critically reviewed and approved the final version for publication.

## Ethics Statement

The study was conducted in accordance with the Declaration of Helsinki and approved by the Ethics Committee of the local university at each study site and was conducted in accordance with the Declaration of Helsinki.

## Conflicts of Interest

The authors declare no conflicts of interest.

## Consent

Informed consent was obtained from all subjects involved in the study.

## Supporting information


**Table S1:** Sample characteristics.
**Table S2:** Univariate analysis of covariance to predict plasma progesterone concentrations by smoking status (smokers versus never‐smokers).
**Table S3:** Univariate analysis of covariance to predict plasma progesterone concentrations by FTND score.
**Table S4:** Univariate analysis of covariance to predict plasma progesterone concentrations by cigarette pack years.
**Table S5:** Univariate analysis of covariance to predict plasma progesterone concentrations by age at smoking onset.
**Table S6:** Univariate analysis of covariance to predict plasma progesterone concentrations by number of cigarettes smoked per day.
**Table S7:** Univariate analysis of covariance to predict plasma progesterone concentrations by exhaled CO.
**Table S8:** Univariate analysis of covariance to predict plasma progesterone concentrations by cotinine concentrations.
**Table S9:** Univariate analysis of covariance to predict plasma progesterone concentrations by QSU score.

## Data Availability

The data that support the findings of this study are available from the corresponding author upon reasonable request.
