# Supplementary material for: No Relevant Associations Between Markers of Smoking Behaviour and Plasma Progesterone Concentrations: Findings From a Sex‐Stratified Cohort Study
Source: Addict Biol. 2025 Aug 8;30(8):e70071. doi: 10.1111/adb.70071 (PMC12334797; doi:10.1111/adb.70071)
Supplement: Supplementary file 1 — Table S1: Sample characteristics. Table S2: Univariate analysis of covariance to predict plasma progesterone concentrations by smoking status (smokers versus never‐smokers). Table S3: Univariate analysis of covariance to predict plasma progesterone concentrations by FTND score. Table S4: Univariate analysis of covariance to predict plasma progesterone concentrations by cigarette pack years. Table S5: Univariate analysis of covariance to predict plasma progesterone concentrations by age at smoking onset. Table S6: Univariate analysis of covariance to predict plasma progesterone concentrations by number of cigarettes smoked per day. Table S7: Univariate analysis of covariance to predict plasma progesterone concentrations by exhaled CO. Table S8: Univariate analysis of covariance to predict plasma progesterone concentrations by cotinine concentrations. Table S9: Univariate analysis of covariance to predict plasma progesterone concentrations by QSU score. [file ADB-30-e70071-s001.docx]

Supplementary Tables:

No Relevant Associations Between Markers of Smoking Behavior and Plasma Progesterone Concentrations: Findings from a Sex-Stratified Cohort Study

Julia Gihl, Norman Zacharias, Sabine Hoffmann, Norbert Thürauf, Gerd Schaller, Georg Winterer, Anne Koopmann, Falk Kiefer, Johannes Kornhuber, Christiane Mühle / Bernd Lenz

| **Supplementary Table 1.** Sample Characteristics | | | | | | | | |
| --- | --- | --- | --- | --- | --- | --- | --- | --- |
|  | *N* | *MeanRF* | *SD* | *N* | *Mean/RF* | *SD* | *T / χ^2^* | *P* |
| *Males 18 – 27 years* | *Smokers* | | | *Never-Smokers* | | | | |
| Age (years) | 103 | 23.69 | 2.49 | 156 | 22.94 | 2.55 | 2.352 | 0.019 |
| BMI (kg/m²) | 103 | 24.12 | 4.15 | 156 | 23.59 | 3.17 | 1.181 | 0.239 |
| Years of education | 103 | 14.96 | 2.27 | 156 | 15.62 | 2.26 | -2.287 | 0.023 |
| AUDIT score | 103 | 6.78 | 3.90 | 156 | 5.11 | 3.38 | 3.654 | <0.001 |
| % Intake of medication | 103 | 21.36 |  | 156 | 29.49 |  | 2.117 | 0.146 |
| FTND score | 101 | 2.42 | 2.63 |  |  |  |  |  |
| Cigarette pack years | 103 | 5.42 | 6.35 |  |  |  |  |  |
| N Cigarettes/d | 103 | 11.83 | 9.55 |  |  |  |  |  |
| CO exhaled (ppm) | 91 | 10.46 | 7.20 | 150 | 1.99 | 1.74 | 11.035 | <0.001 |
| Cotinine (ng/mL) | 103 | 91.60 | 89.97 | 155 | 9.72 | 64.56 | 7.972 | <0.001 |
| QSU score | 102 | 101.59 | 39.81 |  |  |  |  |  |
| Age at smoking onset (years) | 103 | 15.71 | 2.58 |  |  |  |  |  |
| Plasma progesterone (ng/ml) | 103 | 0.494 | 0.132 | 156 | 0.483 | 0.112 | 0.659 | 0.511 |
| *Males 28 – 43 years* | *Smokers* | | | *Never-Smokers* | | | | |
| Age (years) | 102 | 36.00 | 4.68 | 137 | 35.28 | 4.72 | 1.164 | 0.246 |
| BMI (kg/m²) | 102 | 26.20 | 4.70 | 137 | 25.97 | 4.13 | 0.413 | 0.680 |
| Years of education | 102 | 15.58 | 3.06 | 137 | 16.91 | 2.96 | -3.379 | 0.001 |
| AUDIT score | 102 | 5.97 | 4.13 | 137 | 3.25 | 2.35 | 5.971 | <0.001 |
| % Intake of medication | 102 | 28.43 |  | 137 | 35.77 |  | 1.431 | 0.232 |
| FTND score | 100 | 3.33 | 2.54 |  |  |  |  |  |
| Cigarette pack years | 100 | 15.11 | 12.13 |  |  |  |  |  |
| N Cigarettes/d | 102 | 14.83 | 9.58 |  |  |  |  |  |
| CO exhaled (ppm) | 95 | 16.47 | 13.12 | 131 | 1.89 | 1.62 | 10.776 | <0.001 |
| Cotinine (ng/mL) | 101 | 113.98 | 111.19 | 137 | 3.81 | 43.83 | 9.432 | <0.001 |
| QSU score | 98 | 98.76 | 37.19 |  |  |  |  |  |
| Age at smoking onset (years) | 100 | 16.87 | 4.01 |  |  |  |  |  |
| Plasma progesterone (ng/ml) | 102 | 0.434 | 0.131 | 137 | 0.403 | 0.123 | 1.867 | 0.063 |
| *Males 44 – 65 years* | *Smokers* | | | *Never-Smokers* | | | | |
| Age (years) | 143 | 52.50 | 6.00 | 106 | 52.97 | 6.70 | -0.579 | 0.563 |
| BMI (kg/m²) | 143 | 26.71 | 4.22 | 106 | 26.14 | 3.22 | 1.218 | 0.224 |
| Years of education | 143 | 14.34 | 3.22 | 106 | 15.76 | 3.05 | -3.511 | 0.001 |
| AUDIT score | 143 | 4.62 | 3.29 | 106 | 3.09 | 1.99 | 4.541 | <0.001 |
| % Intake of medication | 143 | 39.86 |  | 106 | 38.68 |  | 0.036 | 0.850 |
| FTND score | 140 | 3.64 | 2.65 |  |  |  |  |  |
| Cigarette pack years | 143 | 34.04 | 24.48 |  |  |  |  |  |
| N Cigarettes/d | 143 | 18.49 | 11.89 |  |  |  |  |  |
| CO exhaled (ppm) | 137 | 15.94 | 9.60 | 100 | 1.65 | 1.64 | 17.095 | <0.001 |
| Cotinine (ng/mL) | 143 | 141.95 | 101.97 | 106 | 0.04 | 0.39 | 16.643 | <0.001 |
| QSU score | 139 | 105.65 | 37.16 |  |  |  |  |  |
| Age at smoking onset (years) | 143 | 16.31 | 3.54 |  |  |  |  |  |
| Plasma progesterone (ng/ml) | 143 | 0.360 | 0.121 | 106 | 0.350 | 0.135 | 0.631 | 0.529 |
| Alcohol Use Disorder Identification Test, AUDIT; Body Mass Index, BMI; Fagerström Test for Nicotine Dependence, FTND; N Cigarettes smoked per day, N Cigarettes/d, Questionnaire of Smoking Urges, QSU; Parts per million, ppm. | | | | | | | | |

| **Supplementary Table 2**. Univariate analysis of covariance to predict plasma progesterone concentrations by smoking status (smokers versus never-smokers) | | | | | | | | | |
| --- | --- | --- | --- | --- | --- | --- | --- | --- | --- |
|  | Males 18 – 27 years  (N = 259) | | | Males 28 – 43 years  (N = 239) | | | Males 44 – 65 years  (N = 249) | | |
|  | F | P | partial η^2^ | F | P | partial η^2^ | F | P | partial η^2^ |
| Smoking status | 0.048 | 0.828 | 0.000 | 1.773 | 0.184 | 0.008 | 2.433 | 0.120 | 0.010 |
| Age | 0.522 | 0.471 | 0.002 | 15.833 | <0.001 | 0.065 | 6.818 | 0.010 | 0.028 |
| BMI | 3.514 | 0.062 | 0.014 | 14.837 | <0.001 | 0.062 | 18.335 | <0.001 | 0.072 |
| Years of education | 3.352 | 0.068 | 0.013 | 5.125 | 0.025 | 0.022 | 1.191 | 0.276 | 0.005 |
| AUDIT score | 2.130 | 0.146 | 0.009 | 0.733 | 0.393 | 0.003 | 0.122 | 0.728 | 0.001 |
| Medication | 0.959 | 0.328 | 0.004 | 0.156 | 0.693 | 0.001 | 0.001 | 0.974 | 0.000 |
| Study center | 0.846 | 0.535 | 0.020 | 0.950 | 0.460 | 0.025 | 1.341 | 0.240 | 0.033 |
| Body Mass Index, BMI; Alcohol Use Identification Test, AUDIT. | | | | | | | | | |

| **Supplementary Table 3.** Univariate analysis of covariance to predict plasma progesterone concentrations by FTND score | | | | | | | | | |
| --- | --- | --- | --- | --- | --- | --- | --- | --- | --- |
|  | Males 18 – 27 years  (N = 101) | | | Males 28 – 43 years  (N = 100) | | | Males 44 – 65 years  (N = 140) | | |
|  | F | P | partial η^2^ | F | P | partial η^2^ | F | P | partial η^2^ |
| FTND | 0.639 | 0.426 | 0.007 | 0.029 | 0.865 | 0.000 | 0.000 | 0.995 | 0.000 |
| Age | 0.108 | 0.743 | 0.001 | 8.602 | 0.004 | 0.090 | 7.752 | 0.006 | 0.058 |
| BMI | 5.366 | 0.023 | 0.057 | 4.353 | 0.040 | 0.048 | 8.946 | 0.003 | 0.066 |
| Years of education | 2.198 | 0.142 | 0.024 | 4.828 | 0.031 | 0.053 | 0.165 | 0.685 | 0.001 |
| AUDIT score | 1.499 | 0.224 | 0.017 | 0.573 | 0.451 | 0.007 | 0.000 | 0.996 | 0.000 |
| Medication | 0.071 | 0.790 | 0.001 | 0.025 | 0.875 | 0.000 | 1.230 | 0.270 | 0.010 |
| Study center | 0.874 | 0.518 | 0.056 | 1.239 | 0.295 | 0.079 | 2.394 | 0.032 | 0.102 |
| Fagerström Test for Nicotine Dependence, FTND; Body Mass Index, BMI; Alcohol Use Identification Test, AUDIT. | | | | | | | | | |

| **Supplementary Table 4.** Univariate analysis of covariance to predict plasma progesterone concentrations by cigarette pack years | | | | | | | | | |
| --- | --- | --- | --- | --- | --- | --- | --- | --- | --- |
|  | Males 18 – 27 years  (N = 103) | | | Males 28 – 43 years  (N = 100) | | | Males 44 – 65 years  (N = 143) | | |
|  | F | P | partial η^2^ | F | P | partial η^2^ | F | P | partial η^2^ |
| Cigarette pack years | 0.024 | 0.876 | 0.000 | 0.121 | 0.729 | 0.001 | 0.255 | 0.615 | 0.002 |
| Age | 0.171 | 0.680 | 0.002 | 6.479 | 0.013 | 0.069 | 8.693 | 0.004 | 0.063 |
| BMI | 4.735 | 0.032 | 0.050 | 4.423 | 0.038 | 0.048 | 10.589 | 0.001 | 0.075 |
| Years of education | 2.804 | 0.098 | 0.030 | 4.951 | 0.029 | 0.054 | 0.085 | 0.771 | 0.001 |
| AUDIT score | 1.246 | 0.267 | 0.014 | 0.593 | 0.443 | 0.007 | 0.014 | 0.905 | 0.000 |
| Medication | 0.016 | 0.899 | 0.000 | 0.013 | 0.909 | 0.000 | 1.434 | 0.233 | 0.011 |
| Study center | 0.748 | 0.612 | 0.048 | 1.262 | 0.283 | 0.080 | 2.439 | 0.029 | 0.101 |
| Body Mass Index, BMI; Alcohol Use Identification Test, AUDIT. | | | | | | | | | |

| **Supplementary Table 5.** Univariate analysis of covariance to predict plasma progesterone concentrations by age at smoking onset | | | | | | | | | |
| --- | --- | --- | --- | --- | --- | --- | --- | --- | --- |
|  | Males 18 – 27 years  (N = 103) | | | Males 28 – 43 years  (N = 100) | | | Males 44 – 65 years  (N = 143) | | |
|  | F | P | partial η^2^ | F | P | partial η^2^ | F | P | partial η^2^ |
| Age at smoking onset | 0.191 | 0.663 | 0.002 | 0.000 | 0.997 | 0.000 | 0.001 | 0.979 | 0.000 |
| Age | 0.122 | 0.728 | 0.001 | 8.583 | 0.004 | 0.090 | 8.487 | 0.004 | 0.061 |
| BMI | 5.348 | 0.023 | 0.056 | 4.299 | 0.041 | 0.047 | 10.288 | 0.002 | 0.073 |
| Years of education | 2.979 | 0.088 | 0.032 | 4.585 | 0.035 | 0.050 | 0.166 | 0.685 | 0.001 |
| AUDIT score | 1.211 | 0.274 | 0.013 | 0.561 | 0.456 | 0.006 | 0.002 | 0.961 | 0.000 |
| Medication | 0.016 | 0.899 | 0.000 | 0.031 | 0.861 | 0.000 | 1.288 | 0.259 | 0.010 |
| Study center | 0.765 | 0.599 | 0.049 | 1.253 | 0.288 | 0.080 | 2.546 | 0.023 | 0.105 |
| Body Mass Index, BMI; Alcohol Use Identification Test, AUDIT. | | | | | | | | | |

| **Supplementary Table 6**. Univariate analysis of covariance to predict plasma progesterone concentrations by number of cigarettes smoked per day | | | | | | | | | |
| --- | --- | --- | --- | --- | --- | --- | --- | --- | --- |
|  | Males 18 – 27 years  (N = 103) | | | Males 28 – 43 years  (N = 102) | | | Males 44 – 65 years  (N = 143) | | |
|  | F | P | partial η^2^ | F | P | partial η^2^ | F | P | partial η^2^ |
| Cigarettes/day | 0.790 | 0.377 | 0.009 | 0.102 | 0.750 | 0.001 | 0.139 | 0.710 | 0.001 |
| Age | 0.306 | 0.581 | 0.003 | 8.134 | 0.005 | 0.084 | 8.580 | 0.004 | 0.062 |
| BMI | 6.008 | 0.016 | 0.063 | 4.208 | 0.043 | 0.045 | 10.397 | 0.002 | 0.074 |
| Years of education | 2.130 | 0.148 | 0.023 | 5.356 | 0.023 | 0.057 | 0.115 | 0.735 | 0.001 |
| AUDIT score | 1.102 | 0.297 | 0.012 | 0.601 | 0.440 | 0.007 | 0.010 | 0.920 | 0.000 |
| Medication | 0.047 | 0.829 | 0.001 | 0.002 | 0.965 | 0.000 | 1.375 | 0.243 | 0.010 |
| Study center | 0.847 | 0.537 | 0.053 | 1.296 | 0.267 | 0.080 | 2.448 | 0.028 | 0.102 |
| N Cigarettes smoked per day, Cigarettes/day; Body Mass Index, BMI; Alcohol Use Identification Test, AUDIT. | | | | | | | | | |

| **Supplementary Table 7.** Univariate analysis of covariance to predict plasma progesterone concentrations by exhaled CO | | | | | | | | | |
| --- | --- | --- | --- | --- | --- | --- | --- | --- | --- |
|  | Males 18 – 27 years  (N = 91) | | | Males 28 – 43 years  (N = 95) | | | Males 44 – 65 years  (N = 137) | | |
|  | F | P | partial η^2^ | F | P | partial η^2^ | F | P | partial η^2^ |
| Exhaled CO | 2.482 | 0.119 | 0.031 | 0.208 | 0.649 | 0.003 | 1.512 | 0.221 | 0.012 |
| Age | 0.194 | 0.661 | 0.002 | 8.415 | 0.005 | 0.093 | 6.554 | 0.012 | 0.050 |
| BMI | 5.250 | 0.025 | 0.063 | 2.791 | 0.099 | 0.033 | 12.178 | 0.001 | 0.089 |
| Years of education | 1.569 | 0.214 | 0.020 | 4.700 | 0.033 | 0.054 | 0.069 | 0.793 | 0.001 |
| AUDIT score | 1.275 | 0.262 | 0.016 | 1.239 | 0.269 | 0.015 | 0.000 | 0.985 | 0.000 |
| Medication | 0.000 | 0.999 | 0.000 | 0.011 | 0.917 | 0.000 | 1.356 | 0.247 | 0.011 |
| Study center | 0.776 | 0.591 | 0.056 | 1.237 | 0.296 | 0.083 | 2.611 | 0.020 | 0.112 |
| Body Mass Index, BMI; Alcohol Use Identification Test, AUDIT. | | | | | | | | | |

| **Supplementary Table 8.** Univariate analysis of covariance to predict plasma progesterone concentrations by cotinine concentrations | | | | | | | | | |
| --- | --- | --- | --- | --- | --- | --- | --- | --- | --- |
|  | Males 18 – 27 years  (N = 103) | | | Males 28 – 43 years  (N = 101) | | | Males 44 – 65 years  (N = 143) | | |
|  | F | P | partial η^2^ | F | P | partial η^2^ | F | P | partial η^2^ |
| Cotinine | 1.027 | 0.313 | 0.011 | 0.045 | 0.832 | 0.001 | 2.216 | 0.139 | 0.017 |
| Age | 0.313 | 0.577 | 0.003 | 9.534 | 0.003 | 0.098 | 8.093 | 0.005 | 0.059 |
| BMI | 5.688 | 0.019 | 0.059 | 3.759 | 0.056 | 0.041 | 9.680 | 0.002 | 0.069 |
| Years of education | 2.002 | 0.161 | 0.022 | 5.053 | 0.027 | 0.054 | 0.133 | 0.716 | 0.001 |
| AUDIT score | 1.225 | 0.271 | 0.013 | 0.696 | 0.407 | 0.008 | 0.000 | 0.996 | 0.000 |
| Medication | 0.005 | 0.947 | 0.000 | 0.004 | 0.951 | 0.000 | 1.541 | 0.217 | 0.012 |
| Study center | 0.847 | 0.537 | 0.053 | 1.531 | 0.177 | 0.095 | 2.329 | 0.036 | 0.097 |
| Body Mass Index, BMI; Alcohol Use Identification Test, AUDIT. | | | | | | | | | |

| **Supplementary Table 9.** Univariate analysis of covariance to predict plasma progesterone concentrations by QSU score | | | | | | | | | |
| --- | --- | --- | --- | --- | --- | --- | --- | --- | --- |
|  | Males 18 – 27 years (N = 102) | | | Males 28 – 43 years (N = 98) | | | Males 44 – 65 years (N = 139) | | |
|  | F | P | partial η^2^ | F | P | partial η^2^ | F | P | partial η^2^ |
| QSU score | 0.038 | 0.846 | 0.000 | 0.041 | 0.840 | 0.000 | 0.004 | 0.947 | 0.000 |
| Age | 0.069 | 0.794 | 0.001 | 9.169 | 0.003 | 0.097 | 6.581 | 0.011 | 0.050 |
| BMI | 5.098 | 0.026 | 0.054 | 4.005 | 0.049 | 0.045 | 9.292 | 0.003 | 0.069 |
| Years of education | 3.036 | 0.085 | 0.033 | 4.526 | 0.036 | 0.051 | 0.097 | 0.756 | 0.001 |
| AUDIT score | 1.515 | 0.222 | 0.017 | 0.591 | 0.444 | 0.007 | 0.013 | 0.909 | 0.000 |
| Medication | 0.055 | 0.815 | 0.001 | 0.049 | 0.825 | 0.001 | 1.439 | 0.232 | 0.011 |
| Study center | 0.806 | 0.568 | 0.052 | 1.248 | 0.290 | 0.081 | 2.405 | 0.031 | 0.103 |
| Questionnaire of Smoking Urges, QSU; Body Mass Index, BMI; Alcohol Use Identification Test, AUDIT. | | | | | | | | | |
